# Reply to the Comment on “Sensing Technologies for Extravasation Detection: A Review”

**DOI:** 10.1021/acssensors.3c01182

**Published:** 2023-08-04

**Authors:** Arianna Mazzotta, Ikue Hirata, Pooyan Makvandi, Ilaria Cesini, Chiara Brioschi, Andrea Ferraris, Virgilio Mattoli

**Affiliations:** † Center for Materials InterfacesIstituto Italiano di TecnologiaViale Rinaldo Piaggio 34, Pontedera, PI 56025, Italy; || IIT-BraccoJoint Lab, Istituto Italiano di Tecnologia, 16163 Genova, Italy; ‡ Bracco S.p.A., 20134 Milano, Italy

We recently received and checked your Comment about our latest Review in *ACS Sensors* entitled “Sensing Technologies for Extravasation Detection: A Review”^1^ and we appreciate the interest it has generated in the scientific community.

The authors of the Letter pointed out remarkable observations about the procedure of peripheral intravenous cannulation (PIVC) and cited innovative and recent technologies that practitioners and clinicians are learning to use. Since the main goal of our review is to provide the reader with an overview of the systems and strategies developed over the years to monitor the inserted catheter and possibly detect Intravenous Extravasation (IE) events, we did not go into the details of technologies realized for the placement of the device. Indeed, we believe that these technologies refer to a different topic (improving the placement and insertions of catheters in veins and arteries) and do not really fall within the scope of our paper but could be treated as a separate work, probably worthy of a review paper *per se*.^2^

Some works reported by the Comment’s authors clearly demonstrate the growing interest and awareness of the need to continuously research methods for detection and treatment of extravasation.^3−6^ The cited works are mainly related to the possibility of building a panel of guidelines and best practices for nurses and professionals for the prevention and management of IE events. This aspect is of course extremely important in clinical practice and we thank the authors of the letter for highlighting it, since these tools could be exploited synergistically with the technological devices we presented in our review and could support the human component.

This aspect is also linked to the last comment raised by the Comment’s authors: we are aware that these technologies are not yet ready for clinical use, which is why in the last section Concluding Remarks, Challenges, and Perspectives we provided some considerations on what we believe are important features that an IE prevention device should have. The ultimate goal was not to compare a practitioner’s experience with a technological device in detecting extravasation but to find the best way technology can *help* them with this. Indeed—as the authors of the Comment also mentioned—we should be able to offer to the patient the best method of early detection and identification of peripheral intravenous extravasation.
